# Comment on “Design, synthesis, anticancer activity and molecular docking of quinoline-based dihydrazone derivatives” by J.-X. Lu, H.-R. Lan, D. Zeng, J.-Y. Song, Y.-T. Hao, A.-P. Xing, A. Shen, and J. Yuan, *RSC Adv.*, 2025, **15**, 231–243

**DOI:** 10.1039/d5ra00388a

**Published:** 2025-11-14

**Authors:** Ralf Weiskirchen

**Affiliations:** a Institute of Molecular Pathobiochemistry, Experimental Gene Therapy and Clinical Chemistry (IFMPEGKC), RWTH Aachen University Hospital Aachen Germany rweiskirchen@ukaachen.de +49-(0)241-8088683

## Abstract

This comment addresses significant limitations in the study by Lu *et al.* (*RSC Adv.*, 2025, **15**, 231–243), which investigates the anticancer activity of quinoline-based dihydrazone derivatives. It highlights the misidentification and contamination of cell lines used in the research, specifically BGC-823, BEL-7402, and HL-7702, which are derived from HeLa cells rather than being true representations of human gastric cancer, human hepatoma, or normal liver cells. The letter emphasizes the need for caution when interpreting findings related to cell state (normal *versus* tumor) due to these discrepancies, as they may significantly affect conclusions regarding efficacy and safety profiles in anticancer research.

## Abstract 2

This comment critically evaluates a study by Lu and colleagues, highlighting the misidentification and HeLa cell contamination in the three cell lines used to evaluate the anticancer properties of quinoline-based dihydrazone derivatives.

## Graphical abstract

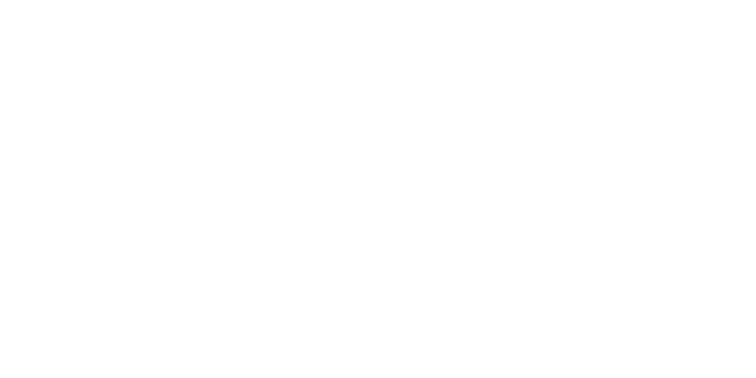

The paper titled “*Design, synthesis, anticancer activity, and molecular docking of quinoline-based dihydrazone derivatives*” by Lu and colleagues discusses the development of a series of quinoline-based dihydrazone derivatives (3a–3d) synthesized from biologically active quinolone.^1^ The authors employed various characterization techniques, including NMR, mass spectrometry, and UV/Vis spectroscopy, to confirm the chemical structures of these compounds.

The anticancer activity of the synthesized derivatives was evaluated against several human cancer cell lines (BGC-823, BEL-7402, MCF-7, and A549) as well as a normal liver cell line (HL-7702). The results indicated that all derivatives exhibited significant anti-proliferative effects, with IC50 values ranging from 7.01 to 34.32 µM. Notably, compounds 3b and 3c demonstrated stronger cytotoxicity against MCF-7 cells compared to the clinically used drug 5-FU. Further investigations revealed that compounds 3b and 3c could induce apoptosis in MCF-7 cells in a dose-dependent manner through mechanisms involving reactive oxygen species (ROS) generation and DNA binding. Molecular docking studies suggested that these compounds interact with cyclin-dependent kinases (CDK2), indicating their potential as CDK inhibitors. Additionally, toxicological assessments showed low toxicity for the synthesized compounds, with no nitrosamine impurities detected. Importantly, the authors stated: “*The results revealed that 3a–3d all had potential anticancer activity while exhibiting no discernible effect on normal liver cells HL-7702*”.^1^ Based on their findings, the authors suggested that quinoline-based dihydrazone derivatives are promising candidates for further development as anticancer agents due to their efficacy and safety profile.

While these findings are intriguing, it is crucial to address significant limitations in this study regarding the cell lines used.

The authors state that BGC-823 is a human gastric cancer cell line; however, although it was originally thought to be a gastric adenocarcinoma, this cell line has been shown to be contaminated by HeLa cells.^2–4^ Similarly, BEL-7402 has also been proven to be contaminated by HeLa and does not represent a human hepatoma cell line as claimed.^2–4^ Most critically, HL-7702, purportedly a normal human liver cell line, is actually derived from HeLa cells. This cell line is also known as L02 or LO-2 and was established at the Shanghai Institute for Cell Biology at the Chinese Academy of Sciences; it was originally thought to originate from normal fetal liver but is now recognized as a derivative of cervical adenocarcinoma HeLa cells.^5^

These misidentified cell lines are listed in the International Cell Line Authentication Committee (ICLAC) register. As of its latest version released on April 26, 2024, this register includes information on 593 cell lines known to be misidentified due to cross-contamination or other mechanisms.^6^ Furthermore, additional details about misidentified cell lines BGC-823 (CVCL_3360), BEL-7402 (CVCL_5492), and HL-7702 (CVCL_6926) can be found in the most recent release of Cellosaurus (release 51 from December 2024), which serves as an important knowledge resource on cell lines used in biomedical research.^7^

Therefore, caution must be exercised when interpreting differences or findings related to the state of the cells (normal *versus* tumor). The potential implications of using contaminated or misidentified cell lines could significantly affect the conclusions drawn regarding anticancer activity and safety profiles of the quinoline-based dihydrazone derivatives.

## Conflicts of interest

The author is a member of the International Cell Line Authentication Committee (ICLAC), which aims to increase the visibility of false or misidentified cell lines. The committee also promotes awareness and authentication testing as effective ways to combat the use of misidentified cells in biomedical research.

## Data Availability

No primary research results, software or code have been included and no new data were generated or analysed as part of this comment.
